# Nutritional Interventions Targeting the Gut Microbiome in MASLD: From Prebiotics and Probiotics to Postbiotics and Fecal Microbiota Transplantation

**DOI:** 10.3390/nu18111765

**Published:** 2026-05-30

**Authors:** Carlo Acierno, Alfredo Caturano, Fannia Barletta, Luca Rinaldi, Ferdinando Carlo Sasso, Luigi Elio Adinolfi, Riccardo Nevola

**Affiliations:** 1 Department of Internal Medicine, San Carlo Hospital, 85100 Potenza, Italy; 2 Department of Medicine and Surgery, University of Basilicata, 85100 Potenza, Italy; 3 Department of Human Sciences and Promotion of the Quality of Life, San Raffaele Roma University, 00166 Rome, Italy; alfredo.caturano@uniroma5.it; 4 Department of Anesthesiology and Intensive Care, San Carlo Hospital, 85100 Potenza, Italy; fannia.barletta@ospedalesancarlo.it; 5 Department of Medicine and Health Science “Vincenzo Tiberio”, Università Degli Studi del Molise, 86100 Campobasso, Italy; luca.rinaldi@unimol.it; 6 Department of Advanced Medical and Surgical Sciences, University of Campania “Luigi Vanvitelli”, 80138 Napoli, Italy; ferdinandocarlo.sasso@unicampania.it (F.C.S.); luigielio.adinolfi@unicampania.it (L.E.A.); 7 Liver Unit, “A. Landolfi” Hospital, AORN S. G. Moscati, 83029 Solofra, Italy; riccardo.nevola@unicampania.it

**Keywords:** MASLD, MASH, gut microbiome, gut–liver axis, prebiotics, probiotics, synbiotics, postbiotics, fecal microbiota transplantation, nutritional interventions

## Abstract

Metabolic dysfunction-associated steatotic liver disease (MASLD) is a highly prevalent liver-centred manifestation of systemic metabolic dysfunction. The gut–liver axis provides a biologically credible therapeutic rationale because intestinal dysbiosis, impaired barrier integrity, microbial metabolites, bile acid signalling, short-chain fatty acids, and trimethylamine N-oxide may influence hepatic steatosis, inflammation, and fibrogenesis. This narrative review critically evaluates dietary patterns, prebiotics, probiotics, synbiotics, postbiotics, and fecal microbiota transplantation (FMT) as microbiome-directed strategies in MASLD. The comparative framework prioritises disease-specific human evidence, clinically meaningful endpoints, trial duration and sample size, reproducibility, safety, and feasibility. Dietary optimisation remains the most clinically grounded intervention, whereas probiotics and synbiotics show modest and heterogeneous signals on biochemical or metabolic surrogate endpoints. Prebiotics are mechanistically coherent but supported by limited liver-centred trials. Postbiotics and microbiome-mediated bioactives remain early-stage and require stricter definitional boundaries. FMT is investigational and should not be extrapolated from its established role in recurrent *Clostridioides difficile* infection. Most available evidence across all intervention categories relies principally on surrogate endpoints—including aminotransferases, insulin resistance indices, lipid parameters, and microbiome compositional shifts—rather than on validated liver-centred outcomes such as histological improvement or quantitative liver fat assessment; this constrains the strength of conclusions that can currently be drawn. Across all categories, microbiome modulation does not by itself establish liver disease modification, and no microbiome-targeted nutritional intervention has yet demonstrated histological benefit in MASLD. Future trials in this field should prioritise validated hepatic endpoints, phenotype-stratified patient enrolment, adequate follow-up duration, and direct comparisons between intervention categories to determine which microbiome-directed strategies, if any, deliver measurable and reproducible hepatic benefit beyond surrogate markers.

## 1. Introduction

### 1.1. Epidemiological Burden and Clinical Relevance of MASLD

Metabolic dysfunction-associated steatotic liver disease (MASLD) has become the dominant form of chronic liver disease worldwide and should be understood not as an isolated hepatic disorder but as a liver-centred expression of systemic metabolic dysfunction [1]. Its global prevalence, commonly estimated at approximately one-third of the adult population [2,3], gives the condition major public health relevance well beyond hepatology, particularly because disease burden is driven not only by progressive steatohepatitis and fibrosis in a clinically important subset, but also by its tight association with obesity, insulin resistance, type 2 diabetes, cardiovascular disease, chronic kidney disease, and hepatocellular carcinoma [4]. Epidemiological data indicate that MASLD patients carry a significantly elevated risk of incident type 2 diabetes, a cardiovascular risk that exceeds liver-related mortality in many cohorts, and a meaningful lifetime risk of hepatocellular carcinoma even in the absence of cirrhosis—underscoring the systemic clinical consequences of the condition [5,6]. This multidimensional risk profile is what makes MASLD clinically consequential: it is highly prevalent, biologically heterogeneous, and embedded within a broader cardiometabolic continuum [5].

The clinical significance of MASLD also derives from its stratified natural history. Although simple steatosis may remain stable in many individuals, a clinically important subgroup progresses to metabolic dysfunction-associated steatohepatitis (MASH), advancing fibrosis, cirrhosis, portal hypertension, liver failure, and liver-related malignancy [6], with fibrosis stage remaining the most robust predictor of long-term liver-specific and all-cause outcomes. At the same time, MASLD cannot be framed solely through hepatic progression, because many patients experience major extrahepatic events before reaching advanced liver stages [7]. For this reason, strategies targeting the gut microbiome have attracted attention not because they offer a proven disease-modifying solution, but because they intersect mechanistically with several of the metabolic, inflammatory, and barrier-related processes that help explain why MASLD behaves as a multisystem disorder [8].

### 1.2. The Nomenclature Transition from NAFLD/NASH to MASLD/MASH: Implications for Clinical Trial Interpretation

The transition from the NAFLD/NASH framework to MASLD/MASH is not merely terminological, because it changes how disease populations are conceptually defined and therefore how older intervention studies should be read [1,9]. The 2023 multisociety consensus repositioned steatotic liver disease within an explicitly metabolic framework, requiring the presence of hepatic steatosis together with at least one cardiometabolic risk factor [1], and thereby aligning nomenclature more closely with the clinical and pathophysiological reality of the disorder [7]. However, most of the microbiome-targeted literature discussed in this review was generated in NAFLD-defined cohorts, often before the current nomenclature and before the broader methodological emphasis on metabolic phenotyping, fibrosis stratification, and endpoint quality [10]. As a result, translation of older evidence into the MASLD era requires caution.

### 1.3. The Gut–Liver Axis as Therapeutic Target: Biological Rationale and Scope of the Review

Interest in the gut microbiome as a therapeutic target in MASLD is biologically well founded [8,11]. The intestine represents a major upstream regulator of hepatic exposure to microbial products, fermentation-derived metabolites, bile acid signalling, and inflammatory stimuli delivered through the portal circulation [12]. The liver receives approximately 70% of its blood supply from the portal vein, which drains the intestinal compartment and delivers a continuous flux of microbial metabolites, bacterial products, and immune signals directly to the hepatic parenchyma [13]. Under conditions of gut dysbiosis, this portal flux becomes enriched with lipopolysaccharide (LPS), secondary bile acids with altered receptor signalling properties, reduced concentrations of short-chain fatty acids (SCFAs), and a variety of microbially derived pro-oxidant and pro-inflammatory mediators [14], promoting lipid accumulation, oxidative stress, mitochondrial dysfunction, and, in susceptible individuals, progressive fibrogenesis [15].

The recognition of the gut–liver axis has generated a structured landscape of microbiome-targeted interventions encompassing prebiotics, probiotics, synbiotics, postbiotics, and fecal microbiota transplantation (FMT) [16]. These categories differ substantially not only in their mechanisms of action but also in the maturity of clinical evidence supporting their use, in the quality of endpoints employed in available trials, and in their feasibility within standard hepatological practice [17]. The established efficacy of fecal microbiota-based therapy in recurrent *Clostridioides difficile* infection (CDI) must not be inappropriately transferred to MASLD [18,19], where the evidentiary base remains far less mature. Recurrent CDI involves a discrete, pathogen-driven disruption of colonisation resistance that FMT can correct through defined ecological reconstitution; MASLD, by contrast, is driven by chronic, multifactorial metabolic and inflammatory dysregulation in which no single microbial target has been validated, making direct analogical transfer of the CDI rationale mechanistically unsound.

### 1.4. Aim and Comparative Thesis of the Review

This review takes a deliberately comparative and clinically disciplined approach. Its central aim is to assess whether microbiome-directed nutritional and ecosystem-level interventions in MASLD are supported by evidence that extends beyond biological plausibility and microbiome compositional change. The review therefore distinguishes direct MASLD/MASH evidence, historically defined NAFLD/NASH evidence likely applicable to MASLD, indirect evidence from obesity, type 2 diabetes, or metabolic syndrome, and preclinical or mechanistic evidence.

The underlying thesis is that the gut microbiome is a credible biological target in MASLD, but the interventions currently proposed to modulate it are not equivalent. Comparative interpretation requires explicit attention to disease-specificity, endpoint strength, trial design, reproducibility, safety, feasibility, and proximity to clinical implementation.

The review is structured around biological rationale, intervention categories, quality of evidence, clinical implications, and research priorities, while avoiding the assumption that microbiome modulation alone constitutes clinically meaningful liver disease improvement.

## 2. Search Strategy and Evidence Selection

### 2.1. Literature Identification

This article was designed as a narrative review rather than as a systematic review or meta-analysis. The literature was identified through targeted searches of PubMed/MEDLINE and through screening of relevant reference lists, using combinations of terms related to MASLD, MASH, NAFLD, NASH, gut microbiome, gut–liver axis, intestinal dysbiosis, intestinal permeability, dietary patterns, prebiotics, probiotics, synbiotics, postbiotics, microbial metabolites, and fecal microbiota transplantation. The search covered publications from approximately 2000 to early 2025, with particular emphasis on studies published after 2015 to reflect the evolving nomenclature, the MASLD/MASH reclassification, and increasingly rigorous methodological standards. Only publications in the English language were considered.

### 2.2. Evidence Prioritisation and Interpretive Hierarchy

Priority was given to consensus documents, clinical practice guidelines, randomised controlled trials, meta-analyses, umbrella reviews, and mechanistic studies with direct relevance to MASLD/MASH or historically defined NAFLD/NASH. Evidence derived from obesity, type 2 diabetes, metabolic syndrome, or preclinical models was considered supportive only when mechanistically relevant and is explicitly interpreted as indirect. Comparative judgments across intervention categories were guided by five predefined dimensions: disease-specificity of the population studied, strength and clinical relevance of the endpoint, consistency of evidence across human studies, safety and tolerability profile, and proximity to clinical implementation. The evidence hierarchy was established a priori as follows: Direct MASLD/MASH evidence was considered the strongest tier. NAFLD/NASH-era human evidence was assigned the next level, given its direct clinical applicability despite nomenclature differences. Evidence extrapolated from adjacent metabolic populations or preclinical models was treated as mechanistically supportive but insufficient to establish clinical conclusions in isolation. This hierarchy reflects the recognition that MASLD/MASH-defined cohorts require explicit metabolic phenotyping not systematically applied in the earlier NAFLD era, that preclinical models cannot fully recapitulate the metabolic heterogeneity and fibrosis background of human MASLD, and that cross-population extrapolation carries inherent uncertainty about mechanistic and therapeutic overlap.

### 2.3. Endpoint Hierarchy and Limits of Narrative Synthesis

For liver-centred interpretation, histological improvement was considered the most stringent endpoint, followed by quantitative liver fat assessment with magnetic resonance imaging-proton density fat fraction (MRI-PDFF), validated non-invasive fibrosis assessment such as vibration-controlled transient elastography (VCTE) or magnetic resonance elastography (MRE), controlled biochemical or metabolic endpoints, and microbiome compositional or metabolomic endpoints. No formal PRISMA-based search strategy, risk-of-bias assessment, or quantitative evidence synthesis was performed. Consequently, the conclusions should be read as calibrated narrative synthesis rather than as formal evidence grading.

## 3. The Gut Microbiome in MASLD: Pathophysiological Framework

The relevance of the gut microbiome in MASLD lies less in reproducible taxonomic signatures than in functional pathways that modify hepatic exposure to microbial products, metabolites, bile acids, and inflammatory signals. This section therefore focuses only on mechanisms directly relevant to nutritional and microbiome-directed interventions: dysbiosis, barrier dysfunction, short-chain fatty acid (SCFA) production, bile acid signalling, trimethylamine N-oxide (TMAO), and host-context dependence [8,20,21].

### 3.1. Compositional and Functional Dysbiosis: Established Findings and Inferential Limits

Human studies consistently suggest that NAFLD/MASLD is associated with compositional and functional perturbations of the gut microbiota, including lower representation of barrier-supportive or SCFA-producing taxa and enrichment of potentially pro-inflammatory communities [22,23,24]. These signals are biologically plausible but not disease-specific: obesity, type 2 diabetes, diet, geography, medication exposure, and sequencing methods strongly influence microbiome composition [25,26]. Accordingly, dysbiosis should be interpreted as a pathophysiological context and potential therapeutic target, not as a stand-alone diagnostic or causal marker.

### 3.2. Intestinal Barrier Dysfunction and Metabolic Endotoxaemia

Barrier dysfunction remains one of the most clinically interpretable links between dysbiosis and hepatic injury. Increased intestinal permeability can permit portal translocation of LPS and other microbial products, activating Toll-like receptor 4 (TLR4) signalling in Kupffer cells, hepatocytes, and hepatic stellate cells and amplifying inflammatory and fibrogenic pathways [12,13,27]. Representative markers of increased intestinal permeability studied in human MASLD cohorts include elevated circulating endotoxin (LPS) concentrations, serum lipopolysaccharide-binding protein (LBP), soluble CD14 (sCD14), and tight junction protein disruption (claudin-1, occludin). Human data support associations between these permeability indices, endotoxaemia, and NAFLD/MASLD severity, but causality and reversibility remain incompletely proven [13,28].

### 3.3. Short-Chain Fatty Acids: Fermentation, Signalling, and Hepatic Implications

SCFAs, particularly butyrate and propionate, provide the most direct mechanistic link between diet, microbial fermentation, epithelial integrity, immune regulation, and hepatic metabolism [29,30]. Butyrate supports colonocyte energetics and barrier function, whereas propionate may influence hepatic gluconeogenesis and de novo lipogenesis [31,32]. In MASLD, reduced SCFA-producing capacity is therefore relevant mainly because it may connect low-fibre dietary patterns, impaired barrier function, metabolic inflammation, and altered substrate handling [33,34].

### 3.4. Bile Acid Metabolism and the FXR/TGR5 Regulatory Axis

Bile acid metabolism is a second intervention-relevant pathway because microbial bile salt hydrolases and 7-alpha-dehydroxylating organisms reshape the bile acid pool and thereby influence farnesoid X receptor (FXR), fibroblast growth factor 19 (FGF19), and TGR5 (Takeda G protein-coupled receptor 5, also known as GPBAR1) signalling [35,36,37,38]. These pathways intersect with hepatic lipid handling, glucose homeostasis, enteroendocrine signalling, and incretin biology, but available human intervention data do not yet allow bile acid remodelling to be used as a validated therapeutic surrogate in MASLD. This limitation reflects several unresolved issues: the composition and concentrations of individual bile acid species vary substantially across MASLD phenotypes and are modified by comorbid diabetes, obesity, and medication exposure; FXR and TGR5 signalling respond to specific bile acid species rather than to total pool size alone; and no intervention trial has demonstrated that normalisation of a bile acid signature independently predicts or mediates histological improvement in MASLD.

### 3.5. Trimethylamine N-Oxide (TMAO) and Choline Metabolism

TMAO, generated from microbiota-derived trimethylamine (TMA) through hepatic oxidation by flavin-containing monooxygenase 3 (FMO3), is associated with adverse metabolic and inflammatory phenotypes in NAFLD/MASLD [39,40,41]. Mechanistically, elevated TMAO has been linked to hepatic lipid accumulation, macrophage inflammatory activation, endoplasmic reticulum stress, and pro-apoptotic signalling in hepatocytes; experimental evidence further suggests that TMAO can modify cytochrome c activity toward a pro-apoptotic peroxidase function, potentially amplifying hepatic injury beyond metabolic effects [40,41]. TMAO levels depend on dietary choline and carnitine intake, the abundance of TMA-lyase-containing microbial taxa, and hepatic FMO3 expression—sources of interindividual variability that complicate the use of TMAO as a standalone therapeutic target. However, its role remains more secure as a candidate mediator or biomarker than as a validated causal treatment target. Claims that nutritional or microbiome-directed interventions improve MASLD through TMAO reduction should therefore require direct liver-centred endpoints, not metabolite change alone.

### 3.6. Inflammatory Amplification, Immune-Metabolic Crosstalk, and Bidirectionality of theGut–Liver Axis

The gut–liver axis is bidirectional. Microbial products and metabolites shape hepatic immune-metabolic signalling, but host metabolic dysfunction also restructures microbial ecology through diet, hyperglycaemia, altered motility, bile acid changes, medication exposure, and adipose inflammation [20,25,26,28]. This reciprocity explains why microbiome signatures often behave as disease-state correlates rather than independent therapeutic targets: observed dysbiosis may be a downstream consequence of metabolic dysfunction as much as an upstream driver of hepatic injury, and interventions that normalise microbiome composition without addressing the underlying metabolic substrate may not produce clinically meaningful hepatic benefit.

### 3.7. Biological Complexity and Heterogeneity: Sex, Diabetes, and MicrobiomeContext-Dependence

Clinical translation is further limited by context-dependence. Sex, type 2 diabetes, fibrosis stage, baseline diet, and medication exposure can modify microbial function and treatment response [25,26]. Precision microbiome approaches—in which patient phenotypes are prospectively characterised and matched with interventions predicted to be effective based on their microbial and metabolic profile—therefore represent an important design direction for future trials in MASLD.

**Figure 1:**
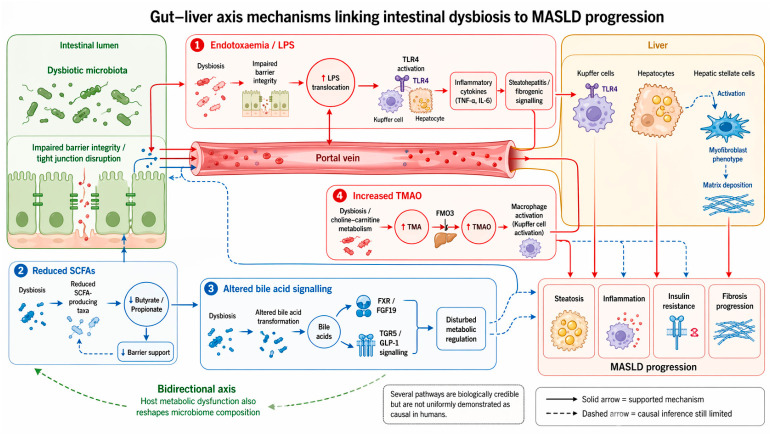
Gut–liver axis mechanisms linking intestinal dysbiosis to MASLD progression. Dysbiosis may influence hepatic steatosis, inflammation, and fibrogenesis through impaired barrier integrity and LPS translocation, altered SCFA production, bile acid–FXR/TGR5 signalling, TMAO generation, and immune-metabolic crosstalk. Solid arrows indicate better-supported human mechanisms; dashed arrows indicate pathways that remain partly inferential in MASLD, **↑** increased.

In synthesis, the gut–liver axis offers a strong biological rationale for intervention, but the causal chain from dysbiosis to treatment response remains only partially demonstrated in humans. The most relevant mechanisms for this review are summarised in Figure 1 and Table 1.

## 4. Dietary Patterns and the Microbiome in MASLD

### 4.1. Diet as the Upstream Modulator of Microbial Ecology in MASLD

Diet is the most clinically grounded microbiome-relevant strategy in MASLD because it modifies substrate availability, microbial ecology, weight, insulin sensitivity, hepatic substrate flux, and inflammatory tone simultaneously [45,46]. The interpretive challenge is attribution: hepatic improvement after dietary intervention may result from weight loss or improved food quality rather than from microbiome mediation per se.

### 4.2. Mediterranean Diet: Mechanistic Plausibility and Clinical Evidence

Among dietary models most relevant to MASLD, the Mediterranean diet remains the most conceptually mature and clinically interpretable [47,48]. Its value lies not in a single nutrient but in a composite nutritional architecture characterised by high intake of plant foods, legumes, whole grains, unsaturated fats, and polyphenol-rich components [45,47]. The green Mediterranean diet—enriched in polyphenols from walnuts and Mankai duckweed—has been shown to produce greater reductions in intrahepatic fat than a conventional Mediterranean diet in a randomised controlled trial, with concomitant changes in gut microbiota composition including expansion of *Akkermansia muciniphila* [49]. This finding is mechanistically suggestive, but the contribution of microbiome remodelling to the hepatic outcome cannot be quantified from available data. In practical terms, the Mediterranean diet has broad and well-documented metabolic relevance in MASLD even when microbial mediation remains only partially resolved [48,50]. Its cardiometabolic benefits—including improvements in insulin sensitivity, lipid profile, blood pressure, and body weight—are supported independently of microbiome-specific effects, and the inability to isolate microbiome-mediated contributions does not diminish the recommendation to adopt Mediterranean dietary principles in MASLD management.

### 4.3. Low-Carbohydrate and Very-Low-Calorie Diets: Metabolic Benefit vs. MicrobiomeTrade-Offs

Low-carbohydrate and very-low-calorie dietary approaches can produce rapid reductions in intrahepatic fat and clear short-term metabolic improvement in MASLD, largely through caloric restriction, reduced substrate delivery to the liver, and suppression of de novo lipogenesis [46]. Their microbiome implications are less straightforward: by reducing intake of fermentable carbohydrates, these diets may diminish microbial diversity and constrain SCFA production, creating a potential trade-off between metabolic efficacy and maintenance of a microbiome configuration generally considered favourable for barrier integrity [45,51]. These approaches illustrate an important principle: not all effective MASLD interventions are microbiologically favourable in the same way, and not all microbiome changes associated with dietary intervention should be assumed to mediate hepatic benefit [47].

### 4.4. Plant-Based Diets and Fermentable Polysaccharides: The Resistant Starch Paradigm

Plant-based dietary patterns and diets enriched in fermentable polysaccharides are of particular interest in MASLD because they align more directly than most other dietary models with the functional logic of microbiome modulation [51,52]. Within this broader category, resistant starch has emerged as a particularly informative model. The most rigorous resistant starch trial in NAFLD demonstrated reductions in intrahepatic triglyceride content measured by magnetic resonance spectroscopy alongside specific microbiome shifts—particularly expansion of *Ruminococcus bromii* and downstream SCFA production [46]—with mechanistic mediation providing one of the clearest examples of how a defined dietary component, a specific microbiome shift, and a liver-centred endpoint can be interpreted within the same experimental framework [33,46].

### 4.5. Time-Restricted Eating and Chrono-Microbiome Dynamics

Time-restricted eating introduces a different dimension to dietary intervention in MASLD by acting not primarily through nutrient composition, but through temporal organisation of feeding and the circadian patterning of host–microbiome interactions [53,54]. Experimental evidence suggests that meal timing can influence rhythmic oscillations in microbial communities and their metabolites, with downstream effects on energy handling, inflammatory signalling, and hepatic metabolic regulation [54]. Accordingly, time-restricted eating should be viewed as a promising but still methodologically unsettled strategy whose microbiome relevance is biologically credible, though not yet sufficient to support strong liver-specific conclusions [45,53].

### 4.6. Confounding by Weight Loss: The Central Methodological Challenge of DietaryMASLD Research

Across dietary interventions, weight loss is the major confounder of microbiome-specific inference. Trials that do not control for energy balance, weight change, and baseline diet cannot support strong claims that microbiome remodelling independently mediates hepatic benefit [45,46,47,51]. An additional challenge is long-term dietary adherence: most microbiome-relevant dietary trials are short, and whether microbiome-mediated benefits persist with sustained dietary modification is insufficiently studied. Weight loss, caloric restriction, and improved dietary quality represent confounders that must be controlled or accounted for before microbiome-specific effects on the liver can be attributed with confidence. Ideally, trials should include isocaloric control arms, objective adherence assessment, and separate reporting of weight-dependent and weight-independent hepatic effects.

The practical conclusion is that dietary optimisation is clinically justified in MASLD, whereas the microbiome-specific contribution to hepatic benefit often remains uncertain. Table 2 summarises dietary patterns according to evidence, microbiome relevance, and limitations.

## 5. Prebiotics

### 5.1. ISAPP Definition and Conceptual Framework: Separating Prebiotics from Dietary Fibre

A disciplined discussion of prebiotics in MASLD must begin with definition, because the term is often used too loosely in both academic and commercial discourse. According to the ISAPP consensus, prebiotics are substrates that are selectively utilised by host microorganisms and confer a health benefit [55]—a definition more restrictive than the broad category of dietary fibre. In the context of MASLD, preserving this definitional boundary is especially important because the literature frequently moves from generic fibre-associated benefit to prebiotic-specific claims without adequately demonstrating selective microbial utilisation [51]. Inulin, fructooligosaccharides (FOS), resistant starches, and related compounds differ substantially in fermentability, ecological selectivity, downstream metabolite profile, and gastrointestinal tolerability [55,56], and treating them as interchangeable weakens the evidentiary coherence of the field.

### 5.2. Mechanistic Basis in MASLD: SCFA Generation, Barrier Reinforcement, and HepaticLipid Metabolism

The mechanistic rationale for prebiotics in MASLD is grounded in their capacity to increase delivery of selectively fermentable substrates to the intestinal microbiota and thereby influence host physiology through downstream metabolite production and mucosal effects [32,51]. The most relevant pathway is enhanced generation of SCFAs, particularly butyrate and propionate, which may reinforce epithelial integrity, reduce translocation of pro-inflammatory microbial products, and modulate hepatic substrate handling through effects on lipogenesis, insulin sensitivity, and immunometabolic signalling [29,33]. Prebiotic-induced fermentation does not produce a single predictable biological output, and hepatic consequences of altered SCFA generation depend on baseline microbiome structure, substrate dose, intestinal transit, host insulin sensitivity, and background diet [30,34].

### 5.3. Clinical Evidence: Trials with Inulin, Fructooligosaccharides, and Resistant Starch

The clinical evidence for prebiotics in MASLD is suggestive but still methodologically limited [57,58]. Trials involving inulin, FOS, inulin-derived formulations, and resistant starch have generally reported modest improvements in liver enzymes, selected metabolic parameters, and, in some studies, quantitative measures of hepatic steatosis [59,60]. A trial of inulin-propionate ester supplementation in adults with NAFLD demonstrated reductions in liver fat by MRI-PDFF and improvements in de novo lipogenesis, with mechanistic data supporting propionate-mediated suppression of hepatic lipid synthesis [60]. The resistant starch trial is among the most informative prebiotic studies in MASLD, providing a plausible causal chain between a defined prebiotic intervention, a specific microbiome shift, and a validated hepatic endpoint [46]. The observation that microbiota-derived succinic acid may mediate attenuating effects on steatohepatitis suggests that the mechanistic spectrum of prebiotic action extends beyond classical SCFA pathways [61]. Key trials have employed inulin and FOS at doses of approximately 10–30 g/day and resistant starch at 15–40 g/day, generally over 8–28 weeks. The most clinically relevant outcomes in these trials have been reductions in alanine aminotransferase (ALT), improvements in liver fat on imaging, and changes in insulin resistance indices; fibrosis-level endpoints have rarely been assessed.

### 5.4. Limitations of the Prebiotic Evidence Base

The limitations of the prebiotic literature in MASLD are structural rather than incidental. Most studies are small and of insufficient duration to assess fibrosis-level outcomes. Endpoint selection is predominantly biochemical or ultrasound-based, without histological validation. The prebiotic category is compositionally diverse—inulin, resistant starch, beta-glucans, pectins, and mixed fibre-based formulations differ substantially in fermentability, downstream metabolite profile, and ecological selectivity [55,56]—and the assumption that different fermentable fibres are interchangeable in their microbiome and hepatic effects is biologically unjustified. Weight loss and caloric restriction are potent confounders of hepatic outcome that are inadequately controlled in many trials [47,51]. A further structural limitation is variability in participants’ baseline diets: in populations with already high habitual fibre intake, the incremental microbiome effect of prebiotic supplementation may be substantially attenuated compared with low-fibre dietary backgrounds. Without baseline dietary characterisation and standardised dietary conditions, inter-trial comparability remains limited.

### 5.5. Safety, Tolerability, and Translational Positioning

From a practical perspective, prebiotics occupy a relatively favourable position because their safety profile is generally benign. The main limitations are tolerability-related rather than safety-critical, with bloating, abdominal discomfort, and altered bowel habit representing the most common constraints on adherence, particularly at higher doses or with rapidly fermentable substrates [51,62]. In comparative terms, prebiotics are best positioned as low-risk, mechanistically credible adjuncts that may complement broader dietary optimisation, rather than as stand-alone liver-directed therapies [47,56].

## 6. Probiotics

### 6.1. Definition, Strain Diversity, and Mechanistic Heterogeneity

Probiotics are defined as live microorganisms that, when administered in adequate amounts, confer a health benefit on the host [63], but in MASLD this category should not be interpreted as a biologically uniform class. Trials have employed single strains, mixed formulations, and high-density multi-strain products across heterogeneous patient populations [64,65], often under the same generic label despite substantial differences in viability, colonisation behaviour, metabolite production, immunomodulatory capacity, and interaction with the resident microbiome [24,66]. This internal heterogeneity is not a secondary technical issue; it is central to how the evidence should be read.

### 6.2. Mechanistic Pathways: Barrier Support, Bile Acid Modulation, Immunomodulation, and Metabolite Production

The mechanistic rationale for probiotics in MASLD rests on several partially overlapping pathways, none of which should be assumed to operate uniformly across strains [16,24]. The most commonly invoked mechanism is support of epithelial barrier integrity, with consequent reduction in bacterial translocation and portal inflammatory signalling [13,67]; this is complemented, in some formulations, by effects on bile acid metabolism and FXR/TGR5-related pathways relevant to hepatic lipid handling [35,38], as well as by modulation of mucosal and systemic immune responses [29,42]. A further layer of interest derives from microbial metabolite production, including SCFAs and other bioactive compounds capable of influencing host metabolism indirectly [30,32].

### 6.3. Clinical Evidence: Meta-Analyses, Umbrella Reviews, and Key RCTs in NAFLD/MASLD

Among microbiome-targeted adjuncts, probiotics have the largest and most structured clinical literature in NAFLD/MASLD, including multiple meta-analyses, umbrella-level syntheses, and a series of randomised controlled trials [68,69]. The umbrella meta-analysis by Musazadeh and colleagues concluded that probiotic supplementation is associated with reductions in serum alanine aminotransferase (ALT) and aspartate aminotransferase (AST) with moderate heterogeneity [64]. Pooled analyses also report modest improvements in insulin resistance indices, lipid parameters, and, less consistently, measures of steatosis [70,71,72]. Abd El Hamid and colleagues reported improvement in the NAFLD Fibrosis Score (NFS) in a probiotic RCT [73], though the clinical significance of NAFLD Fibrosis Score (NFS) changes without histological correlation remains uncertain. The field remains constrained by short treatment duration, heterogeneous products, and the near-complete absence of histological outcomes [74].

### 6.4. Akkermansia muciniphila as a Next-Generation Microbial Candidate

*Akkermansia muciniphila* merits separate consideration because it is more accurately positioned as a next-generation microbial candidate than as a conventional probiotic class effect [75,76]. Its interest in MASLD derives from associations with mucosal integrity, lower endotoxin burden, and favourable metabolic phenotypes [24,75]. However, pasteurised *A. muciniphila* is an inactivated microbial preparation and should not be categorised as a conventional live probiotic, because probiotic definitions require live microorganisms administered in adequate amounts [63]. Evidence from overweight, obese, or type 2 diabetes populations remains indirect for MASLD unless liver-centred endpoints are tested directly [76].

### 6.5. Paediatric Evidence

Paediatric data deserve separate treatment because MASLD in children and adolescents differs from adult disease in developmental context, dietary exposure, microbiome maturation, and phenotype distribution [77,78]. Available studies suggest that probiotic supplementation may improve selected biochemical or imaging-related parameters in paediatric NAFLD/NASH populations [79,80], with early randomised trials reporting directionally favourable effects in obese children and adolescents. However, small sample sizes, heterogeneous formulations, short follow-up, and reliance on surrogate endpoints limit interpretation [77,78]. Probiotic use in paediatric MASLD should therefore be interpreted as an exploratory adjunctive domain rather than a mature therapeutic option.

### 6.6. Limitations: Strain Specificity, Endpoint Immaturity, and Publication Heterogeneity

The limitations of the probiotic evidence base are substantial enough that they define the category as much as its positive signals do. Foremost is strain specificity: products grouped together as probiotics often contain biologically distinct organisms or mixtures whose hepatic relevance cannot be assumed to be equivalent [65,66]. This is compounded by endpoint immaturity, with most trials relying on aminotransferases, metabolic markers, or non-histological assessments of steatosis [64,74]. The heterogeneity of probiotic strains used across trials deserves particular critical attention: Lactobacillus and Bifidobacterium species differ profoundly in their ecological niches, metabolite production profiles, and capacity to influence barrier integrity or bile acid metabolism, yet they are frequently grouped together in meta-analyses as if biologically interchangeable. This strain-level heterogeneity is a fundamental obstacle to evidence synthesis and to the translation of pooled results into product-specific clinical recommendations. Collectively, these factors do not negate the existence of a probiotic signal in MASLD, but they constrain its interpretability and explain why the category cannot support claims stronger than modest adjunctive benefit on surrogate outcomes [69,72].

## 7. Synbiotics

### 7.1. ISAPP Definition: Complementary vs. Synergistic Synbiotics

Synbiotics are defined by the ISAPP as mixtures comprising live microorganisms and substrate(s) selectively utilised by host microorganisms that confer a health benefit [81], and may take two conceptually distinct forms: complementary synbiotics, in which the probiotic and prebiotic components each independently provide benefit; and synergistic synbiotics, in which the prebiotic is specifically designed to selectively nourish the co-administered probiotic strain [81]. This distinction is highly relevant in MASLD, where many studies label combined probiotic–prebiotic formulations as synbiotics without demonstrating true synergism [82,83]. For comparative purposes, synbiotics should not be assumed to represent an inherently more advanced or effective strategy than prebiotics or probiotics alone [84,85].

### 7.2. Clinical Evidence: Meta-Analyses and Key RCTs in NAFLD/MASLD

The clinical literature on synbiotics in NAFLD/MASLD has generated repeatedly positive, though still limited, signals across meta-analyses and randomised trials [82,83,84]. An early pilot randomised trial demonstrated reductions in liver fibrosis markers and ALT after 28 weeks of synbiotic supplementation in NAFLD [86], providing the first signal that synbiotics might influence fibrogenesis-related pathways. The meta-analysis of RCTs by Musazadeh and colleagues (2024) reported statistically significant reductions in ALT, AST, and GGT, along with improvements in triglycerides and inflammatory markers, with moderate-to-high heterogeneity [82]. Further RCTs have confirmed directionally consistent effects in MASLD patients [87,88,89], though heterogeneous formulations, short intervention periods, and near-total reliance on non-histological outcomes limit the evidentiary weight of these findings. A multi-strain synbiotic trial in MASLD also demonstrated improvements in steatosis and microbiome composition [90].

### 7.3. The INSYTE Trial: A Methodological Benchmark and Its Negative Implications

Any balanced assessment of synbiotics in MASLD must give particular weight to the INSYTE trial, because it demonstrated microbiome compositional change without improvement in MRI-PDFF liver fat or convincing fibrosis-related benefit over 12 months [91,92]. This result is methodologically important: it shows that a biologically active microbiome intervention can fail to modify liver-centred outcomes. INSYTE therefore functions as a benchmark against overinterpreting surrogate ecological endpoints.

**Figure 2:**
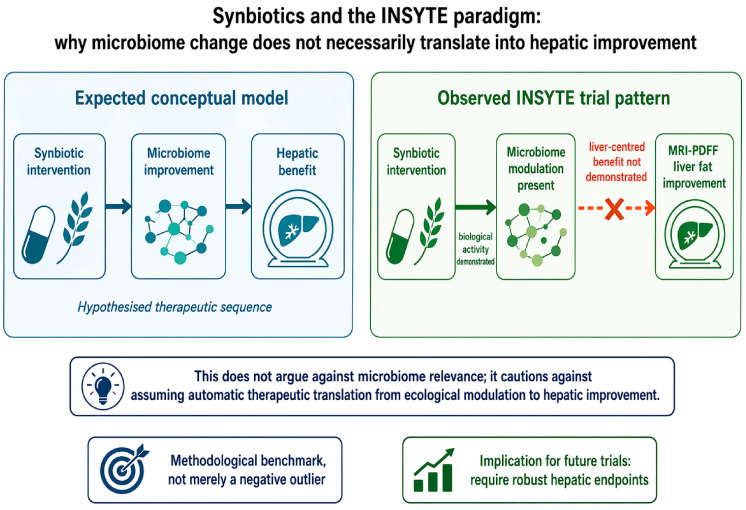
Synbiotics and the INSYTE paradigm. The INSYTE trial showed that microbiome modulation can occur without improvement in MRI-PDFF-quantified liver fat or fibrosis-related outcomes. The figure illustrates the key interpretive principle that ecological change is not automatically equivalent to clinically meaningful hepatic benefit.

The interpretive implications of the INSYTE trial are illustrated in Figure 2.

The principal randomised controlled trials and meta-analyses informing the probiotic and synbiotic literature are summarised in Table 3, with INSYTE highlighted as a negative liver-centred trial rather than as an outlier to be discounted.

### 7.4. Absence of Head-to-Head Comparisons and the Synergism Question

A persistent weakness of the synbiotic literature is the near absence of head-to-head comparisons capable of determining whether combined formulations offer any meaningful advantage over their individual components [82,83]. Without trials that compare synbiotics directly against matched probiotic-only and prebiotic-only arms, claims of synergism remain largely hypothetical. As a result, the category’s apparent promise may reflect cumulative biological plausibility rather than demonstrated superiority [84,85].

### 7.5. Translational Positioning of Synbiotics

In translational terms, synbiotics currently occupy an intermediate position between promise and proof [82,93,94,95]. They are more mechanistically ambitious than prebiotics or many conventional probiotic formulations, and their clinical signal is sufficiently recurrent to justify continued interest; however, they remain constrained by the same endpoint limitations and product heterogeneity that weaken the broader microbiome-intervention literature [78]. Their most defensible current role is as investigational adjuncts with plausible utility in carefully characterised MASLD populations, rather than as evidence-based tools ready for routine clinical incorporation. In direct comparison with probiotics as a reference category, synbiotics do not currently demonstrate a clear superiority in clinical effect: the absence of head-to-head trials designed to detect a difference, combined with the INSYTE negative result for liver fat, prevents any definitive conclusion that combined formulations offer advantages over their individual components for hepatic outcomes in MASLD.

## 8. Postbiotics

### 8.1. ISAPP Definition and Taxonomic Boundaries

The postbiotic field requires strict definitional discipline. In this review, the term postbiotic is reserved for preparations of inanimate microorganisms and/or their components that confer a health benefit, consistent with the 2021 ISAPP consensus [96]. This excludes free microbial metabolites, SCFA prodrugs, polyphenol-derived compounds such as urolithins, hydroxytyrosol, and other microbiome-modulated nutraceuticals, which are discussed here as postbiotic-adjacent or microbiome-mediated bioactives rather than as postbiotics in the strict sense [96,97,98].

The taxonomy adopted in this review is summarised in Table 4.

### 8.2. Mechanistic Rationale and Potential Advantages over Live Organisms

The rationale for true postbiotics in MASLD is the possibility of delivering microbiome-related biological effects without requiring survival, colonisation, or ecological integration of live organisms [96,98]. Potential advantages include greater stability, more standardised manufacturing, and avoidance of live-organism administration in complex patients. These advantages are theoretical unless specific preparations demonstrate liver-centred efficacy.

### 8.3. Clinical Evidence: Butyrate Formulations, Hydroxytyrosol, and SCFA Prodrug Systems

The clinical evidence informing true postbiotics and postbiotic-adjacent strategies in MASLD remains very sparse and heterogeneous [99,100,101]. It is important to emphasise that, at the time of this review, no randomised controlled trial has evaluated a product meeting the strict ISAPP postbiotic definition against a liver histological or validated liver fat endpoint in a MASLD-defined population. Butyrate-based formulations and SCFA delivery systems are biologically coherent because they target microbial metabolite pathways, but most available evidence relies on surrogate endpoints or adjacent metabolic populations [99,100]. Hydroxytyrosol and urolithins are better described as microbiome-mediated nutraceutical or metabolite-related bioactives rather than strict postbiotics; their MASLD relevance should therefore be interpreted through the hierarchy of direct, indirect, and preclinical evidence [101,102]. Similarly, plant-derived multicomponent preparations such as Triphala remain better interpreted as microbiome-adjacent experimental bioactives than as strict postbiotics when evidence is preclinical or indirect [103].

### 8.4. Equol Responsiveness and the Patient Stratification Principle

The concept of equol responsiveness highlights a broader principle that may prove especially important for postbiotic and metabolite-oriented strategies in MASLD [104]: microbiome-related benefit is unlikely to be uniform across patients because the capacity to generate, transform, or respond to specific bioactive compounds is itself biologically stratified. This has direct implications for trial design and interpretation. If only a subset of patients possesses the microbial or metabolic context required to derive benefit from a given postbiotic-related pathway, then conventional unstratified studies may dilute real effects into apparent neutrality [98,104].

### 8.5. Current Maturity and Translational Positioning

At present, postbiotics should be positioned as conceptually promising but clinically immature. Their future relevance depends on adherence to formal definitions, separation from nutraceutical or metabolite-based strategies, and trials linking defined formulations to liver-centred outcomes in biologically characterised MASLD populations [95,96,97,98].

## 9. Fecal Microbiota Transplantation (FMT)

### 9.1. Rationale: Community-Level Ecosystem Reconstitution

Fecal microbiota transplantation (FMT) is the most biologically ambitious microbiome-directed intervention because it attempts community-level ecosystem reconstitution rather than selected nutrient, strain, or metabolite modulation [105,106,107,108]. In MASLD, FMT is best understood as an experimental test of microbiome causality, not as a mature therapeutic option.

### 9.2. Preclinical Plausibility and Inferential Limits

Preclinical transfer experiments and germ-free models support the biological plausibility that microbiota can transmit metabolic phenotypes [105,106]. Their translational value is limited because experimental recipients are highly permissive to engraftment, exposures are controlled, and chronic human MASLD is embedded in diet, adiposity, insulin resistance, and medication context.

### 9.3. Available Human Evidence in NAFLD/MASLD

Direct NAFLD/MASLD evidence remains sparse. The most relevant randomised studies suggest that FMT can modify microbiome composition and may improve small-intestinal permeability, but they have not shown consistent reduction in MRI-PDFF liver fat, insulin resistance, or fibrosis-related outcomes [109,110]. These findings support biological activity, not liver disease modification.

### 9.4. Indirect Evidence from Metabolic Syndrome and Obesity

Evidence from metabolic syndrome and obesity trials is useful but indirect. Lean-donor or capsule-based FMT can transiently improve insulin sensitivity, increase microbial diversity, or promote donor engraftment in selected metabolic populations, particularly when combined with dietary substrates [111,112,113,114]. These effects cannot be assumed to translate into liver-centred benefit in MASLD without dedicated endpoints and longer follow-up.

### 9.5. Donor, Delivery, Safety, and Regulatory Barriers

Clinical translation is constrained by route heterogeneity, donor selection, engraftment durability, stool banking standards, infectious safety, and regulatory uncertainty [106,107,108,115,116]. MASLD imposes a higher risk-benefit threshold than recurrent CDI because it is a chronic non-rescue indication. Donor screening must therefore include both infectious risk control and metabolic phenotyping, and trial protocols must standardise stool processing, dosing, delivery route, diet co-intervention, and follow-up duration. Long-term safety data on FMT in metabolic (non-CDI) indications remain limited: post-authorisation experience from CDI programmes has identified rare but serious transmissible adverse events, including drug-resistant organism transfer, and regulatory agencies have issued enhanced screening requirements in response. Whether these risks apply proportionally to metabolic MASLD applications—where treatment is elective, chronic, and involves recipients with immune-metabolic comorbidities—cannot be assumed. The absence of standardised FMT protocols for MASLD, including variation in stool preparation method, delivery route (colonoscopic, enema, nasojejunal, or oral capsule), donor selection criteria, engraftment monitoring approaches, and repeat-dosing schedules, represents a fundamental barrier to inter-trial comparability and to regulatory advancement. Practical methodological challenges in donor selection include identifying metabolically lean, microbiome-diverse donors without metabolic syndrome comorbidities, obtaining adequate stool volume for banking, ensuring consistent engraftment, and managing donor drop-out over the course of longitudinal trial programmes.

### 9.6. Lessons from CDI and Trial-Only Status in MASLD

The success of FMT in recurrent Clostridioides difficile infection (CDI) should not be extrapolated to MASLD [18,19]. CDI is driven by loss of colonisation resistance against a defined pathogen, whereas MASLD involves chronic, multifactorial metabolic and inflammatory pathways without a single microbial target. Current guidance and available MASLD evidence support FMT only within clinical trials, with liver-centred endpoints and rigorous safety governance [18,95,107].

**Figure 3:**
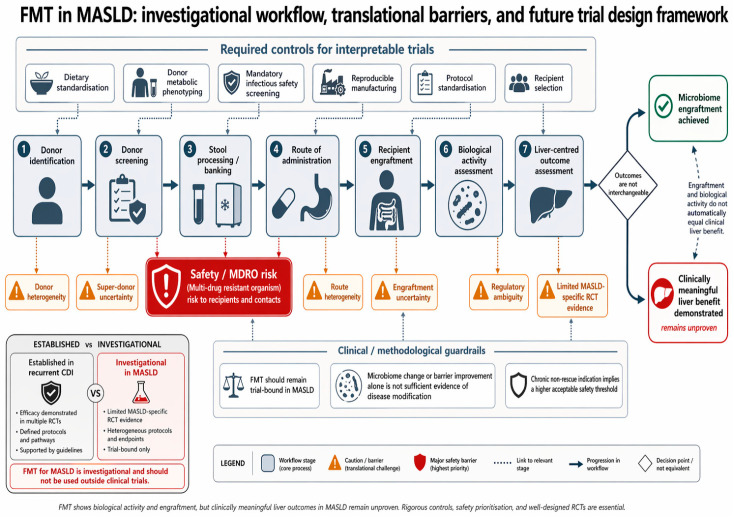
Fecal microbiota transplantation in MASLD: mechanistic probe rather than translational therapy. FMT tests whether community-level microbiome reconstitution can modify gut-liver signalling, but current MASLD evidence is limited by sparse RCTs, uncertain engraftment durability, donor and route heterogeneity, safety concerns, and regulatory complexity.

The rationale, limitations, and trial-only positioning of FMT in MASLD are summarised in Figure 3.

## 10. Comparative Synthesis and Clinical Implications

### 10.1. Explicit Comparative Framework Across Intervention Categories

The intervention categories reviewed here were compared using the evidence framework defined above: disease-specificity, endpoint strength, human trial volume and consistency, safety, feasibility, and translational readiness [17,94,95]. Dietary patterns remain the most clinically grounded domain because they are already embedded in MASLD management, even when microbiome mediation is difficult to isolate. Probiotics and synbiotics have the largest human adjunctive literature, but most signals remain modest and surrogate-based. Prebiotics are biologically coherent but less mature. Postbiotics and FMT remain investigational, with FMT carrying the greatest operational and safety constraints.

A defensible translational maturity ranking is therefore: dietary patterns > probiotics/synbiotics > prebiotics > postbiotics > FMT. This ranking describes evidentiary maturity and clinical deployability, not proven liver disease modification. No category has demonstrated histological disease modification in MASLD [117].

**Figure 4:**
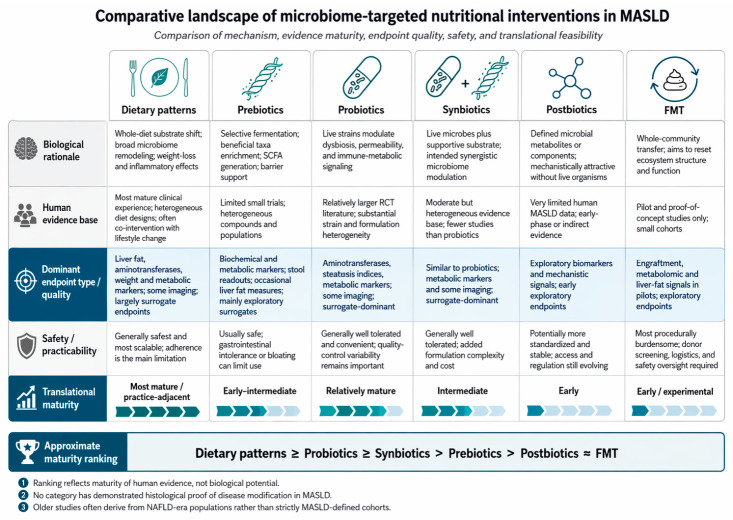
Comparative landscape of microbiome-targeted interventions in MASLD. The figure provides a conceptual overview of evidence maturity, mechanistic plausibility, safety, and clinical deployability across dietary patterns, prebiotics, probiotics, synbiotics, postbiotics, and FMT. Chevron bars indicate the relative translational maturity of each intervention, ranging from early/experimental to practice-adjacent. Operational evidence criteria are detailed separately in Table 5.

The comparative landscape of microbiome-targeted interventions is illustrated in Figure 4, while the evidence hierarchy and translational maturity of each category are summarised in Table 5.

### 10.2. Endpoint Quality Across Categories: The Universal Limitation

Across intervention classes, endpoint quality remains the dominant limitation. Aminotransferases, insulin resistance indices, lipid parameters, inflammatory markers, microbiome composition, and metabolomic shifts indicate biological activity but cannot by themselves establish meaningful improvement in steatohepatitis or fibrosis trajectory [17,94,117]. Future trials should prioritise MRI-PDFF, validated fibrosis assessment, and histology when clinically justified.

### 10.3. Safety and Practicability Profile of Each Category

Safety and feasibility also separate these approaches. Dietary intervention is broadly scalable and aligned with standard care. Prebiotics, probiotics, and synbiotics are generally low-risk but limited by product heterogeneity and tolerability. Postbiotics may eventually offer manufacturing advantages but remain under-validated. FMT has the least favourable safety–practicability profile because it requires donor screening, regulated processing, route-specific delivery, and long-term surveillance.

### 10.4. Translational Maturity Ranking and Interpretive Principles for Clinical Practice

The available evidence supports a cautious translational ranking in which dietary patterns occupy the most usable clinical position, followed by probiotics and synbiotics as the most evidence-developed adjunctive microbiome-targeted categories, with prebiotics somewhat behind but still biologically coherent, and postbiotics and FMT remaining investigational [94,95]. The main clinical principle that follows is conservative: microbiome-targeted interventions in MASLD may be discussed as adjunctive strategies within a broader metabolic framework, but none currently justifies being presented as a disease-modifying therapy in its own right [17,117]. On a practical clinical level, the following guidance can be offered based on current evidence: dietary optimisation toward Mediterranean-style patterns, weight management, and cardiometabolic risk factor control represent the evidence-based foundation of MASLD care and are fully compatible with standard hepatological management. Probiotic supplementation may be considered as an optional adjunct in motivated patients with appropriate tolerance, with the explicit understanding that current evidence supports only modest surrogate benefits and not histological disease modification; no specific strain can be recommended on the basis of proven hepatic benefit. Synbiotics occupy a similar evidence position to probiotics. Prebiotics as dietary supplements represent a low-risk adjunct for patients tolerating them. Postbiotics and FMT should be restricted to clinical trial settings.

On current data, it is reasonable to conclude that some microbiome-targeted interventions, particularly probiotics and synbiotics, can generate modest improvements in biochemical or metabolic domains relevant to MASLD [72,82], and that dietary models with favourable microbial effects are fully compatible with standard management [46,49]. It is not reasonable to infer from this that specific products should be routinely recommended for liver disease control, nor that more complex strategies such as postbiotics or FMT have earned a place outside investigational settings [18,96].

### 10.5. Clinical Practice Implications

The key clinical implications for practice are summarised in Box 1.

Box 1 **What clinicians can and cannot infer from current evidence.**

- Dietary optimisation, weight management, and cardiometabolic risk reduction remain the clinically justified foundation of MASLD care.
- Microbiome modulation should not be interpreted as liver disease modification unless accompanied by validated liver-centred improvement.
- Probiotics and synbiotics may show modest surrogate biochemical or metabolic benefits, but current evidence does not justify their use as disease-modifying MASLD therapies.
- Prebiotics are mechanistically plausible and generally low risk, but they remain adjunctive dietary tools rather than validated liver-directed treatment.
- Postbiotics and microbiome-mediated bioactives require stricter definition, product standardisation, and MASLD-specific trials.
- FMT should not be used for MASLD outside clinical trials because direct hepatic benefit is unproven and safety, donor, route, and regulatory issues remain unresolved.

### 10.6. Contextual Integration with Pharmacological Therapy: GLP-1 Receptor Agonists and Resmetirom

GLP-1 receptor agonists and resmetirom provide clinical context rather than the focus of this review. These therapies act through more clearly validated metabolic or liver-directed pathways, and microbiome-targeted nutritional strategies should not be presented as substitutes for pharmacological therapy when the latter is indicated [118]. Broader nutritional and alcohol-related metabolic exposures remain part of this integrated context [119]. Whether microbiome modulation can improve response, tolerability, or residual metabolic risk in combination with these agents remains plausible but unproven.

## 11. Limitations of This Review

This narrative review is limited by the absence of a PRISMA-based systematic retrieval strategy, formal risk-of-bias assessment, or quantitative meta-analysis. The available literature is heterogeneous with respect to nomenclature, patient phenotype, fibrosis stage, intervention definition, formulation, dose, duration, comparator, and endpoint selection. Most intervention studies were conducted under NAFLD/NASH rather than MASLD/MASH terminology, and evidence from obesity, type 2 diabetes, metabolic syndrome, or preclinical models remains indirect. These limitations justify the cautious interpretive stance maintained throughout the review.

## 12. Conclusions and Future Directions

### 12.1. Endpoint Quality: The First Priority for Future Trials

The most urgent requirement for progress in microbiome-targeted MASLD research is improvement in endpoint quality. At present, much of the literature is built on surrogate measures that are useful for detecting biological activity but insufficient for establishing clinically meaningful disease modification. Future trials will need to adopt stronger liver-centred outcome architectures, including rigorous quantitative imaging (MRI-PDFF) and validated non-invasive fibrosis assessment (VCTE), and, where justified, histological endpoints, if microbiome-targeted strategies are to move from plausible adjuncts to genuinely evaluable therapeutic candidates in MASLD. The endpoint standards already established in pharmaceutical NASH and MASLD trials provide the benchmark that nutritional and microbiome-directed studies must increasingly approach.

### 12.2. Intervention Standardisation and Heterogeneity Reduction

A second priority is reduction of intervention heterogeneity. Across prebiotics, probiotics, synbiotics, postbiotics, and FMT, the literature is weakened by inconsistent definitions, variable formulation quality, poorly harmonised dosing strategies, and limited comparability across trials [64,82,107]. Strain standardisation, preparation consistency, transparent reporting of probiotic and prebiotic formulation details, and explicit control for weight loss as a co-primary variable must become normative [55,81]. Without better standardisation, the field will continue to generate literature that is expandable in volume but only partially cumulative in meaning [66].

### 12.3. Patient Stratification and Precision Microbiome Approaches

A third major requirement is better patient stratification. MASLD is biologically heterogeneous, and the same is true of the intestinal microbiome; it is therefore increasingly implausible that unselected populations will respond uniformly to microbiome-targeted interventions. Differences in obesity, insulin resistance, diabetes status, fibrosis stage, dietary background, sex, and baseline microbial function are likely to influence both the biological relevance of dysbiosis and the probability of treatment response [5,26]. Sex-stratified trial designs and analyses are specifically warranted given the reported differences in microbiome composition and functional capacity between male and female MASLD patients [25]. Precision microbiome approaches therefore remain aspirational, but already represent a more credible developmental trajectory than continued reliance on undifferentiated trial populations.

### 12.4. FMT Programme Design for MASLD: A Proposed Research Framework

If FMT is to remain part of the MASLD research agenda, its development will require a far more structured framework than the one reflected in the current literature. Any future programme should be explicitly trial-bound, built around rigorous donor metabolic phenotyping in addition to infectious safety screening, standardised stool processing and banking procedures, controlled dietary background, and recipient selection criteria capable of enriching for biologically plausible responders. Repeated assessment should be anchored to liver-centred endpoints rather than microbiome change alone, so that ecological engraftment is interpreted as a mechanism to be tested rather than as a surrogate of success. This should be understood as a proposed research framework, not a clinical pathway: without such design discipline, FMT is unlikely to generate interpretable evidence in MASLD at all.

### 12.5. Microbiome-Targeted Interventions as Adjuncts to Pharmacotherapy

The most realistic future role for microbiome-targeted interventions in MASLD is likely to be adjunctive rather than substitutive. As pharmacological options with stronger liver-centred evidence emerge—including GLP-1 receptor agonists and resmetirom [118]—the relevant question is no longer whether microbiome modulation should compete with drug therapy, but whether it can enhance response, improve metabolic context, or address residual pathophysiological domains not fully captured by existing agents. This possibility is biologically plausible, particularly for interventions that influence barrier function, inflammatory tone, or microbial metabolite signalling, yet it remains unproven.

### 12.6. Final Synthesis: From Plausibility to Evidence-Calibrated Translation

The gut microbiome has evolved from a speculative explanatory framework in MASLD to a scientifically legitimate domain for therapeutic investigation; however, it has not yet yielded a consolidated treatment paradigm supported by validated liver-centred evidence [8,120]. The evidence reviewed here demonstrates biological plausibility and selected surrogate benefits across multiple intervention categories, but does not yet establish clinically meaningful liver disease modification for any microbiome-directed nutritional strategy. Meaningful progress will require moving beyond demonstrating microbiome modulation per se and toward proving that defined interventions, administered to phenotypically appropriate patients at the right disease stage, produce measurable and reproducible hepatic benefit by validated endpoints. Until that evidentiary threshold is achieved, microbiome-targeted strategies should be positioned as adjunctive and investigational rather than as disease-modifying therapies for MASLD.

### 12.7. Future Clinical Perspectives

Several near-term clinical translational priorities emerge from this review. First, dietary intervention frameworks—particularly Mediterranean-style patterns—should be prioritised in clinical practice as the most evidence-based, scalable, and safe microbiome-relevant strategy. Second, adequately powered randomised controlled trials of probiotic and synbiotic formulations with validated non-invasive or histological liver endpoints are urgently needed to determine whether biochemical improvements translate to liver benefit. Third, precision approaches that match patients by microbiome profile, metabolic phenotype, and fibrosis stage to specific interventions represent the most scientifically promising research direction. Fourth, the integration of microbiome-targeted strategies with pharmacological therapies (GLP-1 receptor agonists, resmetirom) should be examined in appropriately designed combination trials. Fifth, FMT programmes for MASLD require standardised protocols with liver-centred primary endpoints, rigorous donor metabolic phenotyping, and long-term safety follow-up before any broader clinical consideration can be justified.

## Figures and Tables

**Table 1:** Key pathophysiological mechanisms of the gut–liver axis in MASLD.

Mechanism	Key Mediators	Hepatic Consequences	References
Metabolic endotoxemia and LPS translocation	LPS, TLR4, claudin-1, occludin, zonulin	Activation of Kupffer cells and hepatocytes; increased release of TNF-α, IL-6, and IL-1β; amplification of necroinflammatory injury.	[12,13,27]
Deficiency of short-chain fatty acids	Butyrate, propionate, acetate; *Faecalibacterium prausnitzii* and other butyrate-producing taxa	Impaired intestinal barrier integrity; increased hepatic de novo lipogenesis; insulin resistance; promotion of hepatic steatosis.	[29,30,33,34]
Dysregulated bile acid metabolism	DCA, LCA, FXR, TGR5, FGF19, CYP7A1	Impaired FXR-mediated suppression of bile acid synthesis; reduced GLP-1 secretion; altered lipid handling and hepatic metabolic homeostasis.	[35,36,37,38]
TMAO overproduction	Trimethylamine lyases, FMO3, choline, carnitine	Hepatic lipid accumulation; macrophage inflammatory activation; potential contribution to steatohepatitis progression.	[39,40,41]
Microbiota-driven immune-metabolic crosstalk	Microbial PAMPs, inflammasome activation, macrophage polarisation	Systemic and hepatic inflammation; hepatic stellate cell activation; fibrogenesis.	[8,20,42,43,44]
Bidirectional host–microbiome disruption	Hyperglycemia, dietary excess, adipose tissue inflammation	Perpetuation of dysbiosis by host metabolic dysfunction, reinforcing gut–liver axis injury and disease progression.	[20,25,26,28]

Abbreviations: LPS, lipopolysaccharide; TLR4, Toll-like receptor 4; TNF-α, tumour necrosis factor-alpha; IL-6, interleukin-6; IL-1β, interleukin-1beta; DCA, deoxycholic acid; LCA, lithocholic acid; FXR, farnesoid X receptor; TGR5, Takeda G protein-coupled receptor 5 (GPBAR1); FGF19, fibroblast growth factor 19; CYP7A1, cytochrome P450 7A1; TMAO, trimethylamine N-oxide; TMA, trimethylamine; FMO3, flavin-containing monooxygenase 3; PAMP, pathogen-associated molecular pattern; MASLD, metabolic dysfunction-associated steatotic liver disease.

**Table 2:** Dietary patterns and microbiome relevance in MASLD: clinical evidence summary.

Dietary Pattern	Main Microbiome-Related Effect	Key Clinical Evidence	Main Limitations	References
Mediterranean diet	Increased microbial diversity; expansion of *Akkermansia muciniphila* in the green Mediterranean diet model.	DIRECT-PLUS RCT showed greater reduction in intrahepatic fat with the green Mediterranean diet compared with the conventional Mediterranean diet; TANGO RCT supported the relevance of an Asian-adapted Mediterranean-style dietary pattern.	The specific contribution of microbiome modulation to hepatic benefit cannot be quantified; effects are confounded by weight loss, caloric restriction, and overall improvement in diet quality.	[48,49,50]
Low-carbohydrate diets/very-low-calorie diets	Potential reduction in microbial diversity and decreased short-chain fatty acid production, particularly with restrictive dietary patterns.	Associated with rapid reduction in hepatic fat and improvement in metabolic parameters, largely driven by caloric restriction and weight loss.	Possible microbiome trade-offs; limited evidence from sustained, liver-centred RCTs assessing long-term hepatic and microbiome outcomes.	[46,51]
Plant-based diets and fermentable polysaccharides/resistant starch	Expansion of *Ruminococcus bromii* and increased production of butyrate, succinate, and other microbial fermentation products.	MRS-based evidence of reduced intrahepatic triglyceride content; one high-quality trial supports a mechanistic sequence linking resistant starch intake, microbiome remodeling, and liver fat reduction.	Evidence is mainly driven by a single high-quality trial; short duration; findings may depend on the specific resistant starch type, dose, and background diet.	[46,52]
Time-restricted eating	Potential restoration of circadian microbial oscillations and rhythmic short-chain fatty acid production.	Preclinical NASH models support microbiome–circadian effects; pilot clinical data suggest possible metabolic benefit.	Insufficient liver-centred RCT evidence; hepatic interpretation remains largely inferential and partly extrapolated from preclinical or early clinical data.	[53,54]

Abbreviations: MASLD, metabolic dysfunction-associated steatotic liver disease; NASH, non-alcoholic steatohepatitis; RCT, randomised controlled trial; MRI-PDFF, magnetic resonance imaging–proton density fat fraction; DIRECT-PLUS, Dietary Intervention Randomised Controlled Trial Polyphenols Unprocessed Study; TANGO, Trial Assessing Glycemic Outcomes in Asian-adapted Mediterranean Diet; SCFA, short-chain fatty acid.

**Table 3:** Key randomised controlled trials and meta-analyses of probiotics and synbiotics in NAFLD/MASLD.

Study	Category	Intervention	Duration	Key Findings	Ref.
Musazadeh et al., 2022	Probiotics; umbrella meta-analysis	Probiotic supplementation versus placebo/control in NAFLD/MASLD.	Variable across included studies.	Probiotic supplementation was associated with reductions in ALT and AST, although with moderate heterogeneity and no histological confirmation of liver disease modification.	[64]
Zhou et al., 2023	Probiotics; meta-analysis	Multiple probiotic strains versus control in NAFLD.	Variable across included studies.	Reported significant reductions in ALT and AST, with modest improvements in selected metabolic parameters.	[68]
Abd El Hamid et al., 2024	Probiotics	Probiotic supplementation in NAFLD.	Not reported.	Improvement in NAFLD Fibrosis Score was observed, but without histological correlation or direct confirmation of fibrosis regression.	[73]
Zhang et al., 2025	Inactivated microbial preparation/postbiotic candidate	Pasteurised *Akkermansia muciniphila* versus placebo in individuals with type 2 diabetes and overweight.	12 weeks.	Reduced body weight and HbA1c, with directionally favorable hepatic signals; MASLD-specific liver endpoints require confirmation.	[76]
Eslamparast et al., 2014	Synbiotics	Inulin plus multi-strain probiotic versus placebo in NAFLD.	28 weeks.	Reduced ALT and selected fibrosis-related markers in a pilot study with limited sample size.	[86]
Musazadeh et al., 2024	Synbiotics; meta-analysis	Synbiotic supplementation versus control in NAFLD/MASLD.	Variable across included studies.	Associated with reductions in ALT, AST, GGT, and triglycerides, although with substantial heterogeneity.	[82]
Scorletti et al., 2020—INSYTE	Synbiotics	Inulin plus multi-strain probiotic versus placebo in NAFLD.	12 months.	Demonstrated microbiome modulation without reduction in MRI-PDFF-assessed liver fat; the primary liver-fat endpoint was negative.	[91]
Dinavari et al., 2026	Synbiotics	Multi-strain synbiotic plus lifestyle intervention versus placebo/control in MASLD.	16 weeks.	Reported reduction in steatosis grade together with improvement in microbiome composition.	[90]

Abbreviations: NAFLD, non-alcoholic fatty liver disease; MASLD, metabolic dysfunction-associated steatotic liver disease; ALT, alanine aminotransferase; AST, aspartate aminotransferase; GGT, gamma-glutamyl transferase; MRI-PDFF, magnetic resonance imaging–proton density fat fraction; HbA1c, glycated haemoglobin A1c; NFS, NAFLD Fibrosis Score; INSYTE, Investigating the effects of a SYnbiotic on liver fat and fibrosis in non-alcoholic fatty liver disease (trial name); HOMA-IR, homeostatic model assessment of insulin resistance.

**Table 4:** Taxonomy of microbiome-related intervention categories relevant to MASLD.

Category	Definition Used in This Review	Examples Relevant to Manuscript	Interpretive Consequence	References
Conventional probiotics	Live microorganisms that confer a health benefit when administered in adequate amounts.	*Lactobacillus*/*Bifidobacterium*-containing formulations; multi-strain live products.	Effects are strain- and formulation-specific; class-level claims are weak.	[63,64,65,66]
Next-generation live biotherapeutics	Live, rationally selected organisms or consortia developed as defined therapeutic candidates.	Live *Akkermansia muciniphila* or defined microbial consortia, when viable and standardised.	Require product-specific MASLD trials and regulatory characterisation.	[75,76,95]
Inactivated microbial preparations/true postbiotics	Inanimate microorganisms and/or components conferring health benefit.	Pasteurised *Akkermansia muciniphila*; heat-killed or lysed microbial preparations.	Not conventional probiotics; safety and mechanism differ from live organisms.	[76,96,97,98]
Microbial metabolites/delivery systems	Defined metabolites or prodrugs related to microbial function but not microorganism preparations.	Butyrate formulations, SCFA prodrugs, succinate-related pathways.	Mechanistically relevant but not postbiotics under strict ISAPP terminology.	[61,96,99,100]
Microbiome-adjacent nutraceuticals	Host- or diet-derived bioactives whose effects may be mediated partly through microbiome remodelling.	Hydroxytyrosol, urolithins, polyphenol-derived compounds.	Should be interpreted as indirect or adjunctive evidence unless MASLD endpoints are directly tested.	[98,101,102,103]

Abbreviations: ISAPP, International Scientific Association for Probiotics and Prebiotics; MASLD, metabolic dysfunction-associated steatotic liver disease; SCFA, short-chain fatty acid.

**Table 5:** Comparative evidence hierarchy and translational maturity of microbiome-targeted interventions in MASLD.

Category	Evidence Base	Strongest Liver-Centred Endpoint	Main Limitations	Clinical Interpretation	References
Dietary patterns	Direct MASLD/NAFLD human evidence, supported by broad metabolic and lifestyle data.	MRI-PDFF or quantitative liver fat in selected dietary trials; metabolic endpoints.	Microbiome mediation often inseparable from weight loss, caloric restriction, and improved food quality.	Foundation of MASLD care; not a microbiome-specific therapy.	[45,46,47,48,49,50,53,54]
Prebiotics	Mostly NAFLD-era trials plus mechanistic SCFA and barrier data.	ALT, metabolic markers, and selected imaging-based steatosis outcomes.	Small trials, short duration, heterogeneous substrates, limited fibrosis data.	Low-risk adjunct; not a validated liver-directed treatment.	[55,56,57,58,59,60,61,62]
Probiotics	Largest adjunctive NAFLD/MASLD human literature, but strain/formulation-specific.	ALT/AST, insulin resistance, lipid markers; limited imaging; no histological proof.	Short trials, heterogeneous products, inconsistent reproducibility.	Investigational adjunct; product-specific evidence required.	[63,64,65,66,67,68,69,70,71,72,73,74,75,76]
Synbiotics	Human RCTs and meta-analyses; INSYTE provides high-quality null liver-fat evidence.	Biochemical/metabolic endpoints; INSYTE negative for MRI-PDFF liver fat.	True synergism rarely proven; many trials small and formulation-specific.	Investigational adjunct; superiority over components unproven.	[81,82,83,84,85,86,87,88,89,90,91,92]
Postbiotics/microbiome-mediated bioactives	Strong consensus definitions but sparse MASLD-specific human evidence.	Ultrasound steatosis or metabolic markers in limited studies; many data remain preclinical or indirect.	Definitional instability, product diversity, limited liver-centred trials.	Investigational; requires strict taxonomy and defined MASLD endpoints.	[96,97,98,99,100,101,102,103,104]
FMT	Sparse direct NAFLD/MASLD RCT evidence; indirect metabolic syndrome/obesity evidence.	Barrier permeability and engraftment; no consistent MRI-PDFF or fibrosis benefit.	Donor, route, processing, safety, regulatory, and durability constraints.	Clinical-trial only; not appropriate for routine MASLD care.	[18,19,105,106,107,108,109,110,111,112,113,114,115,116]

Abbreviations: MASLD, metabolic dysfunction-associated steatotic liver disease; NAFLD, non-alcoholic fatty liver disease; RCT, randomised controlled trial; MRI-PDFF, magnetic resonance imaging–proton density fat fraction; VCTE, vibration-controlled transient elastography; MRE, magnetic resonance elastography; FMT, fecal microbiota transplantation; SCFA, short-chain fatty acid; ALT, alanine aminotransferase; AST, aspartate aminotransferase; GGT, gamma-glutamyl transferase; INSYTE, Investigating the effects of a SYnbiotic on liver fat and fibrosis (trial name).

## Data Availability

No new data were created or analysed in this study. Data sharing is not applicable to this article.

## Abbreviations

ALT	alanine aminotransferase
AST	aspartate aminotransferase
CDI	*Clostridioides difficile* infection
DCA	deoxycholic acid
FGF19	fibroblast growth factor 19
FMT	fecal microbiota transplantation
FMO3	flavin-containing monooxygenase 3
FOS	fructooligosaccharides
FXR	farnesoid X receptor
GGT	gamma-glutamyl transferase
GLP-1 RA	glucagon-like peptide-1 receptor agonist
HOMA-IR	homeostatic model assessment of insulin resistance
ISAPP	International Scientific Association for Probiotics and Prebiotics
LCA	lithocholic acid
LPS	lipopolysaccharide
MASLD	metabolic dysfunction-associated steatotic liver disease
MASH	metabolic dysfunction-associated steatohepatitis
MRI-PDFF	magnetic resonance imaging–proton density fat fraction
NAFLD	non-alcoholic fatty liver disease
NASH	non-alcoholic steatohepatitis
NFS	NAFLD Fibrosis Score
RCT	randomised controlled trial
SCFA	short-chain fatty acid
TLR4	Toll-like receptor 4
TMA	trimethylamine
TMAO	trimethylamine N-oxide
VCTE	vibration-controlled transient elastography
MRE	magnetic resonance elastography
